# Plastome structure, phylogenomics, and divergence times of tribe Cinnamomeae (Lauraceae)

**DOI:** 10.1186/s12864-022-08855-4

**Published:** 2022-09-08

**Authors:** Tian-Wen Xiao, Xue-Jun Ge

**Affiliations:** 1 Key Laboratory of Plant Resources Conservation and Sustainable Utilization, South China Botanical Garden, Chinese Academy of Sciences, Guangzhou, China; 2 Center of Conservation Biology, Core Botanical Gardens, Chinese Academy of Sciences, Guangzhou, China

**Keywords:** Lauraceae, Plastome, Hypervariable region, Divergence time estimation

## Abstract

**Background:**

Tribe Cinnamomeae is a species-rich and ecologically important group in tropical and subtropical forests. Previous studies explored its phylogenetic relationships and historical biogeography using limited loci, which might result in biased molecular dating due to insufficient parsimony-informative sites. Thus, 15 plastomes were newly sequenced and combined with published plastomes to study plastome structural variations, gene evolution, phylogenetic relationships, and divergence times of this tribe.

**Results:**

Among the 15 newly generated plastomes, 14 ranged from 152,551 bp to 152,847 bp, and the remaining one (*Cinnamomum chartophyllum* XTBGLQM0164) was 158,657 bp. The inverted repeat (IR) regions of XTBGLQM0164 contained complete *ycf2*, *trnI*^*CAU*^, *rpl32*, and *rpl2*. Four hypervariable plastid loci (*ycf1*, *ycf2*, *ndhF*-*rpl32*-*trnL*^*UAG*^, and *petA*-*psbJ*) were identified as candidate DNA barcodes. Divergence times based on a few loci were primarily determined by prior age constraints rather than by DNA data. In contrast, molecular dating using complete plastid protein-coding genes (PCGs) was determined by DNA data rather than by prior age constraints. Dating analyses using PCGs showed that *Cinnamomum* sect. *Camphora* diverged from *C.* sect. *Cinnamomum* in the late Oligocene (27.47 Ma).

**Conclusions:**

This study reports the first case of drastic IR expansion in tribe Cinnamomeae, and indicates that plastomes have sufficient parsimony-informative sites for molecular dating. Besides, the dating analyses provide preliminary insights into the divergence time within tribe Cinnamomeae and can facilitate future studies on its historical biogeography.

**Supplementary Information:**

The online version contains supplementary material available at 10.1186/s12864-022-08855-4.

## Background

Tribe Cinnamomeae (Lauraceae), named by Baillon in 1870, includes *Cinnamomum*, *Phoebe*, *Machilus*, *Alseodaphne*, *Persea*, *Nothaphoebe*, *Apollonias*, *Hufelandia*, *Nesodaphne*, *Haasia*, *Beilschmiedia*, *Aiouea*, and *Potameia* [1]. Kostermans [2] reclassified Lauraceae and placed *Ocotea*, *Cinnamomum*, *Actinodaphne*, *Sassafras*, *Umbellularia*, *Dicypellium*, *Aiouea*, *Aniba*, *Endlicheria*, *Licaria*, *Urbanodendron*, *Systemonodaphne*, and *Phyllostemonodaphne* in tribe Cinnamomeae based on inflorescence traits and cupule structures. However, tribe Cinnamomeae was dismantled by van der Werff and Richter [3], and genera of this tribe were placed in tribe Perseeae and tribe Laureae according to inflorescence traits and wood and bark anatomical structures. Many other studies also used different character combinations and even chemical constituents to revise this tribe and its related groups [4–6] and drew distinct conclusions attributed to convergent or parallel evolution of morphologies in Lauraceae and the fact that different biologists assigned different weights to morphologies in taxonomy [7, 8]. The difficulties in morphology-based taxonomy and the development of molecular phylogenetics have promoted the transition from traditional to phylogeny-based classification of Lauraceae [9].

In the past decades, evolutionary biologists have made much progress in the phylogenetics of tribe Cinnamomeae, but the relationships within the tribe have not been fully resolved. The phylogenetic tree based on *matK* indicated the monophyly of the *Cryptocarya* group, the *Chlorocardium*-*Mezilaurus* clade, and the *Persea* group [10]. However, the relationships of tribes Cinnamomeae and Laureae remained unresolved due to insufficient informative sites. The phylogenetic tree based on ITS showed that tribes Cinnamomeae and Laureae were monophyletic, and *Sassafras* and *Umbellularia* should be excluded from tribe Laureae and placed in tribe Cinnamomeae [11]. However, phylogenetic relationships within tribes were unclear. Huang et al. [12] comprehensively sampled the *Cinnamomum* group, reconstructed the tree of tribe Cinnamomeae using ITS + *LEAFY* + *RPB2*, and found that *Aiouea* was sister to *Cinnamomum* sect. *Cinnamomum* + *Kuloa*. Unfortunately, *Sassafras* and the *Ocotea* complex in the New World were not included. Penagos Zuluaga et al. [13] used restriction site-associated DNA sequencing (RAD-seq) data and constructed a highly resolved maximum likelihood (ML) tree of *Aiouea* and the *Ocotea* complex, but the other clades of tribe Cinnamomeae were not sampled. Plastid phylogenomics showed that *Nectandra* + *Ocotea* were sisters to all the other clades of tribe Cinnamomeae [14, 15], which was in conflict with the nuclear-loci-based tree of Huang et al. [12]. Phylogenetic conflicts between plastid and nuclear data are common in plants and typically accepted as a result of uniparental (plastid) inheritance versus biparental (nuclear) inheritance [16, 17].

Tribe Cinnamomeae consists of shrubs or trees and is the most species-rich tribe of Lauraceae with more than 1000 species [6]. Most species are distributed in the tropical rainforests and subtropical evergreen broad-leaved forests of Asia and the Americas, with a small number in Oceania and Africa [6]. Ecological prominence and wide and disjunctive distributions make this tribe an ideal target for studying historical biogeography. Divergence time estimation is the foundation for biogeographic studies. However, several studies used few loci and neglected the potential impact of limited informative sites on divergence time estimations (e.g., [12, 18, 19]). Brandley et al. [20] suggested that divergence times were primarily determined by prior age constraints rather than DNA data when informative sites were insufficient. Divergence times of the *Cinnamomum* group were estimated using only three nuclear loci that contained limited informative sites [12], and therefore, they need reinvestigation.

In general, the complete plastid genomes (plastomes) contain more informative sites than several nuclear or plastid loci; therefore, plastome phylogenomics can better resolve the phylogenetic relationships of plants. With the rapid development of next-generation sequencing, plastomes became cost-effective and have been widely used to explore plant evolution [21]. To date, 48 plastomes representing 29 species of tribe Cinnamomeae have been reported in GenBank and Lauraceae Chloroplast Genome Database (LCGDB; https://lcgdb.wordpress.com/) (accessed on 20 March 2022), which accounts for only ca. 2.3% of the total species diversity. Hence, we report 15 newly sequenced plastomes of tribe Cinnamomeae and combine them with published plastomes (Table S1), aiming to: (1) explore plastome structural variations, (2) identify hypervariable regions as promising DNA barcodes for future study, (3) assess the influence of limited parsimony-informative (Pi) sites on divergence time estimation, and (4) reestimate the divergence time using plastomes.

## Materials and methods

### Sampling, DNA extraction, and sequencing

In this study, 15 samples were used for DNA sequencing. These samples represented 14 species from two sections (sect. *Camphora* and sect. *Cinnamomum*) in the genus *Cinnamomum*. Materials were collected from living plants in the field and botanical gardens. Plants were identified and deposited as voucher specimens in the herbarium of the South China Botanical Garden, Chinese Academy of Sciences (IBSC) (Table S2). The cetyltrimethylammonium bromide (CTAB) method [22] was used to extract genomic DNA of each sample from silica gel-dried leaf tissues. The DNA concentration was measured with the Qubit 3.0 Fluorometer dsDNA HS Assay Kit (Invitrogen, Carlsbad, CA, USA), and DNA fragment size distribution was assessed using 1% agarose gel electrophoresis. The library with an insert size of 270 bp was constructed at the Beijing Genomics Institute (BGI; Shenzhen, China). Paired-end reads of 150 bp were sequenced by genome skimming with the HiSeq X Ten system (Illumina Inc., San Diego, CA, USA).

### Plastome assembly and annotation

Low-quality reads and adaptors were removed using Trimmomatic v0.36 [23], and FastQC [24] was used to assess data quality. About 2 Gb clean reads were obtained for each sample. The plastomes were assembled using NOVOPlasty v2.7.2 [25] and GetOrganelle v1.7.5.3 [26]. To ensure that the plastomes were correctly assembled, the clean reads were mapped to plastomes using Burrows-Wheeler Aligner v0.7.17-r1188 [27] and SAMtools v1.9 [28], and the results were manually checked in the Geneious v9.1.3 [29]. The plastomes were annotated using the GeSeq–Annotation of Organellar Genomes program (https://chlorobox.mpimp-golm.mpg.de/geseq.html) [30]. Thereafter, the start and stop codon positions of protein-coding genes (PCGs) were checked and adjusted in Geneious. Raw reads and newly generated plastomes were submitted to GenBank (accession numbers shown in Table S2). Plastome maps were drawn using the online program OrganellarGenomeDRAW tool (OGDRAW; https://chlorobox.mpimp-golm.mpg.de/OGDraw.html) [31].

### Comparative genomic analyses and hypervariable regions

For the 15 newly sequenced plastomes, rearrangement and inversion were detected with Mauve v1.1.1 [32] in Geneious. The expansion and contraction of boundaries between inverted repeat (IRa and IRb) regions and single copy (LSC and SSC) regions were identified using IRscope v0.1 [33]. To validate the IR boundary variation, primers were designed in Geneious, and polymerase chain reaction (PCR) and gel electrophoresis experiments were performed.

To detect variable regions across tribe Cinnamomeae, a 39-plastome dataset was created comprising 30 species of *Cinnamomum*, one species of *Nectandra*, seven species of *Ocotea*, and one species of *Sassafras* (Table S1). Genome variability was assessed using mVISTA [34] under Shuffle-LAGAN mode, with *Cinnamomum osmophloeum* (GenBank accession number: MT384386) randomly selected as a reference. The 39 plastomes were aligned using MAFFT [35] with default settings and nucleotide diversity (Pi) was calculated in DnaSP v5 [36], with window length and step size set as 1000 and 250 bp, respectively. Variations in Pi across sites were plotted using ggplot2 [37] in R v4.0.4 [38].

### Repeat sequence identification

For the 39-plastome dataset, three types of repetitive sequences, including dispersed repeats, simple sequence repeats (SSRs), and tandem repeats, were examined. For dispersed repeats (including forward, reverse, complement, and palindromic repeats), the REPuter online program (https://bibiserv.cebitec.uni-bielefeld.de/reputer) was used with default settings: maximum computed repeats = 50 and minimal repeat size = 8 [39]. To determine SSRs, the MIcroSAtellite identification tool (MISA v2.1) [40] was used with default settings: the minimum number of repetitions for mono-, di-, tri-, tetra-, penta-, and hexanucleotides was 10, 6, 5, 5, 5, and 5, respectively. To detect tandem repeats, Tandem Repeats Finder v4.09 [41] was used with the following criteria: matching weight = 2, mismatching penalty = 7, indel penalty = 7, minimum alignment score = 80, maximum period size = 500, match probability = 80, and indel probability = 10.

### Phylogenetic analyses

Three concatenated sequence matrices were prepared for phylogenetic analyses: (1) complete plastomes with one IR removed to reduce redundancy (CP-c); (2) protein-coding genes (PCG-c); and (3) non-protein-coding genes (NPCG-c), including intergenic regions, tRNAs, rRNAs, and introns. Because gaps can influence tree topology [42, 43], sites with more than 50% gap percentage were trimmed using ClipKIT [44]. The three matrices consisted of 11 plastomes from tribe Laureae as outgroups, and 43 plastomes from tribe Cinnamomeae, representing 30 species of *Cinnamomum*, one species of *Nectandra*, seven species of *Ocotea*, and one species of *Sassafras* (Table S1). All loci were extracted using the Python script PersonalUtilities (https://github.com/Kinggerm/PersonalUtilities) and were aligned using MAFFT with default settings. The alignments were manually checked in Geneious and were concatenated using AMAS v1.0 [45]. Alignment lengths, number of variable sites, number of parsimony-informative sites, and GC content of CP-c, PCG-c, and NPCG-c were summarized using AMAS [45]. The best-scoring ML tree was searched in RAxML v8.2.11 [46] with the GTRGAMMA model and 1000 bootstrap replicates, and by specifying the rapid bootstrapping strategy (‘-f a’ option).

### Selective pressure analyses

To detect genes under positive selection, selective pressure analyses were performed on extracted PCGs using CODEML in PAML 4.9j [47] following the protocol of Xiao et al. [48]. The PCG-c ML tree was used as input, with bootstra*p* values and branch length removed using MEGA X [49]. Site-specific model comparisons (M3 vs. M0, M2a vs. M1a, M8 vs. M7) were invoked to identify positively selected sites [50], and the likelihood ratio test (LRT) was performed in R. Nucleotide sites with Bayes empirical bayes (BEB) value > 0.95 and *p* value < 0.05 were considered positively selected.

### Effect of uninformative loci on molecular dating

To assess the effect of uninformative loci on divergence time estimation, two molecular dating analyses were conducted in BEAST v2.6.3 [51]. First, three nuclear loci (ITS, *LEAFY*, *RPB2*; Huang et al. [12]) were downloaded from GenBank (Table S3). These loci were aligned using MAFFT, and the alignments were concatenated into a matrix using AMAS. The best-fitted substitution model (GTR + I + G4) was determined in ModelTest-NG [52] according to the Akaike information criterion (AIC). The GAMMA distribution model (G4) accounts for rate heterogeneity among sites and works sufficiently well for most datasets [51]. Two secondary calibration points (stem and crown ages of the *Cinnamomum* group) and one fossil calibration point (stem age of *Alseodaphne*) with normal distributions were used for prior age constraints following Huang et al. [12]. Subsequently, molecular dating analysis (hereafter: full analysis) was performed for 100,000,000 generations, sampling every 10,000 generations. Second, “Sample From Prior” was selected and other parameters were kept unchanged in BEAUTi, generating a new configuration file for another molecular dating analysis without DNA data (hereafter: prior-only analysis).

After completing the two dating analyses, the distributions and mean of posterior age of the splitting time of *Aiouea* and *C.* sect. *Cinnamomum* + *Kuloa* were compared. If the distributions and mean of divergence time estimated from DNA data (full analysis) were similar to the prior-only analysis, then the estimated times were concluded to only (or mainly) be influenced by prior age constraints rather than by DNA data.

### Molecular dating using PCGs

To estimate divergence times within tribe Cinnamomeae, newly sequenced plastomes were combined with published plastomes from GenBank and LCGDB, generating a 100-plastome dataset. This dataset represented 39 species of tribe Cinnamomeae, 12 species of tribe Laureae, 17 species of tribe Perseeae, three species of tribe Caryodaphnopsideae, three species of tribe Neocinnamomeae, 17 species of tribe Cryptocaryeae of Lauraceae, two species of Hernandiaceae, and three species of Calycanthaceae (Table S1).

The best-fitted model (GTR + I + G4) was selected for the PCGs dataset in ModelTest-NG according to AIC. The uncorrelated relaxed log-normal molecular clock allows sequence evolutionary rate to vary among different parts of a phylogeny [16], and also accounts for uncertainties in phylogenetic relationships and fossil calibrations [17], thus was used in this study. Yule model was specified for the speciation process. GAMMA distribution was set for the prior of birthrate, and the exponential distribution was assigned for the prior of ucldMean and ucldStdev. The BEAST analysis was run for 400,000,000 Markov chain Monte Carlo (MCMC) generations with the sampling frequency of 40,000.

Because fossils attributed to *Cinnamomum* are unreliable [12], four macrofossils of the outgroups were used for node calibrations. First, *Virginianthus calycanthoides* Friis et al. is a well-preserved fossil flower from the early to middle Albian of Cretaceous [53], and the fossil can be used to calibrate the crown age of Laurales [54]. Here, a log-normal distribution was set for the crown node of Laurales with offset, mean, and standard deviation as 107.1, 0.5, and 0.6, respectively. Second, *Potomacanthus lobatus* von Balthazar et al. is a charcoalified fossil flower described from the early to middle Albian of Cretaceous, and this fossil was used to calibrate the stem node of Lauraceae with a log-normal distribution and offset of 106.8, mean of 0.5, and standard deviation of 0.6, following Kondraskov et al. [55]. Third, *Neusenia tetrasporangiata* Eklund is a flower bud fossil described from the Santonian/Campanian (ca. 83 ma) of Cretaceous, and it shows a close relationship to extant *Neocinnamomum* based on its psilate pollen [56, 57]. This fossil was used to calibrate the crown node of the *Neocinnamomum*-*Caryodaphnopsis*-core Lauraceae clade by specifying a log normal distribution with an offset of 83, a mean of 1, and a standard deviation of 1.1. Fourth, *Machilus maomingensis* Tang et al. is a leaf fossil described from the late or middle Eocene, and it exhibits a close similarity to extant *Machilus* based on leaf architecture and cuticle [58]. This fossil was used to calibrate the stem node of *Machilus*, assigning a log-normal distribution with an offset of 33.7, a mean of 1, and a standard deviation of 0.85. To ensure that the estimated times were determined by DNA data rather than by prior age constraints, an additional BEAST analysis was performed by specifying “Sample From Prior” with 100,000,000 MCMC generations and sampling frequency of 10,000, while the other parameters were unchanged.

Tracer v1.7.1 [59] was to confirm the convergence of parameters (ESS ≥ 200). After discarding the first 20% of posterior trees as burn-in, TreeAnnotator in BEAST v2.6.3 was used to generate the maximum clade credibility tree [51].

## Results

### Plastome features

All 15 newly sequenced plastomes shared a typical quadripartite structure—LSC, SSC, IRa, and IRb. The genome size of *Cinnamomum chartophyllum* XTBGLQM0164 was 158,657 bp, substantially larger than the other 14 *Cinnamomum* plastomes ranging from 152,551 bp (*C. cassia* D053) to 152,847 bp (*C. austrosinense*) (Table 1). The size of the IR region of *C. chartophyllum* was 25,974 bp, approximately 5000 bp larger than the other 14 samples (20,060–20,132 bp). The size of the SC region of *C. chartophyllum* XTBGLQM0164 was smaller than the other 14 samples. All 15 plastomes had 79 unique PCGs, 30 unique tRNAs, and four unique rRNAs. However, the *C. chartophyllum* XTBGLQM0164 plastome had 85 PCGs, 37 tRNAs, and eight rRNAs, and the other 14 plastomes had only 82 PCGs, 36 tRNAs, and eight rRNAs (Tables 1 and S4). The GC content of the 15 plastomes ranged from 39.1 to 39.2%.

**Table 1:** Summary of the 15 newly sequenced plastomes of tribe Cinnamomeae

Taxa	Voucher	Plastome size (bp)	LSC (bp)	IR (bp)	SSC (bp)	GC content (%)	Number of PCGs (unique)	Number of tRNAs (unique)	Number of rRNAs (unique)
*Cinnamomum appelianum* Schewe	CFL3846	152,748	93,725	20,128	18,767	39.1	82 (79)	36 (30)	8 (4)
*Cinnamomum austrosinense* H. T. Chang	2,520,043	152,847	93,758	20,092	18,905	39.2	82 (79)	36 (30)	8 (4)
*Cinnamomum burmannii* Blume	XTBGLQM0487	152,740	93,691	20,074	18,901	39.2	82 (79)	36 (30)	8 (4)
*Cinnamomum cassia* Presl	D053	152,551	93,617	20,066	18,802	39.2	82 (79)	36 (30)	8 (4)
*Cinnamomum cassia* Presl	FZ013	152,773	93,725	20,066	18,916	39.2	82 (79)	36 (30)	8 (4)
*Cinnamomum chartophyllum* H. W. Li	XTBGLQM0164	158,657	87,867	25,974	18,842	39.1	85 (79)	37 (30)	8 (4)
*Cinnamomum glanduliferum* Nees	CFL2920	152,702	93,604	20,114	18,870	39.1	82 (79)	36 (30)	8 (4)
*Cinnamomum iners* Reinw. ex Bl.	XTBGLQM0484	152,741	93,702	20,060	18,919	39.2	82 (79)	36 (30)	8 (4)
*Cinnamomum longepaniculatum* N. Chao ex H. W. Li	wh020	152,722	93,599	20,132	18,859	39.1	82 (79)	36 (30)	8 (4)
*Cinnamomum pauciflorum* Nees	CFL3983	152,703	93,711	20,074	18,844	39.1	82 (79)	36 (30)	8 (4)
*Cinnamomum pingbienense* H. W. Li	XTBGLQM0740	152,628	93,683	20,074	18,797	39.2	82 (79)	36 (30)	8 (4)
*Cinnamomum rufotomentosum* K. M. Lan	CFL2798	152,727	93,605	20,132	18,858	39.1	82 (79)	36 (30)	8 (4)
*Cinnamomum septentrionale* Hand.-Mazz.	HZ105	152,726	93,640	20,114	18,858	39.1	82 (79)	36 (30)	8 (4)
*Cinnamomum tamala* T. Nees & Nees	XTBGLQM0255	152,774	93,697	20,074	18,929	39.2	82 (79)	36 (30)	8 (4)
*Cinnamomum tenuipile* Kosterm.	XTBGLQM0666	152,725	93,685	20,074	18,892	39.2	82 (79)	36 (30)	8 (4)

### IR expansion and contraction, and genome rearrangement

**Fig. 1:**
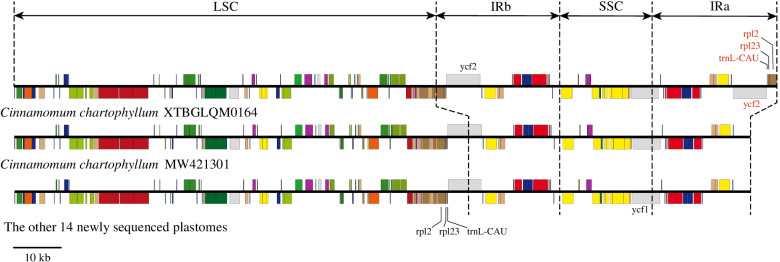
Gene maps of newly sequenced plastomes and *Cinnamomum chartophyllum* MW421301. Genes related to inverted repeat (IR) expansion are colored in red (*ycf2*, *trnL*^*CAU*^, *rpl23*, and *rpl2* of *Cinnamomum chartophyllum* XTBGLQM0164)

*Cinnamomum chartophyllum* harbored double complete *trnI*^*CAU*^, *rpl32*, *rpl2*, and *ycf2* in the IR regions, showing significant IR expansion (Figs. 1 and S1). To ensure that the expansion was not caused by sequencing or assembly errors, two pairs of primers were designed in Geneious, targeting *rpl2* exon2, *trnH*^*GUG*^, and their intergenic region (Table S5). *C. cassia* D053 and *C. longepaniculatum* wh020 were selected as a comparison for PCR and gel electrophoresis experiments. The experimental result showed that the targeting region existed in *C. chartophyllum* (Fig. S2), but not in the other species, suggesting significant IR expansion in the *C. chartophyllum* plastome. Besides, according to the Mauve analysis, no rearrangement and inversion were detected in the 15 plastomes (Fig. S3).

### Hypervariable regions

**Fig. 2:**
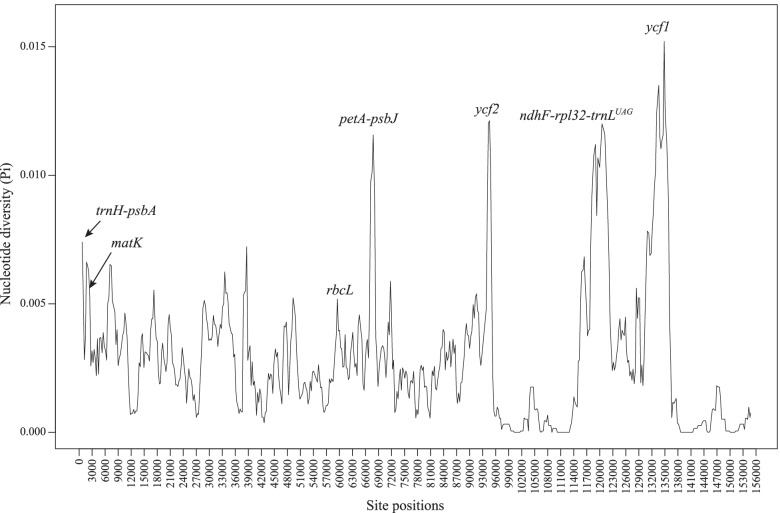
The variation of nucleotide diversity across 39 plastomes of tribe Cinnamomeae. Four hypervariable loci (*ycf1*, *ndhF*-*rpl32*-*trnL*^*UAG*^, *ycf2*, and *petA*-*psbJ*) and three standard DNA barcodes (*trnH*-*psbA*, *matK*, and *rbcL*) are indicated

Genome variability analysis using mVISTA showed that sequence divergence within tribe Cinnamomeae was mostly located in the intergenic regions and two PCGs, *ycf1* and *ycf2* (Fig. S4). According to the nucleotide diversity analysis, four loci with higher Pi values were *ycf1*, *ycf2*, *ndhF*-*rpl32*-*trnL*^*UAG*^, and *petA*-*psbJ* (Fig. 2). Besides, three universal barcoding loci (*trnH*-*psbA*, *matK*, and *rbcL*) are shown in Fig. 2. The Pi values of *trnH*-*psbA*, *matK*, and *rbcL* were substantially lower than the four hypervariable loci.

### Characterization of repetitive sequences

A total of 1950 dispersed repeats were detected for the 39 species, of which forward, palindromic, and reverse repeats constituted the majority (95.18%), and complement repeats constituted the minority (4.82%) (Table S6). The number of forward repeats (716) was higher than palindromic (568) or reverse (572) repeats. The lengths of dispersed repeats were similar within *Cinnamomum* and *Sassafras* (18–87 bp), but were smaller than *Nectandra* and *Ocotea* (18–275 bp). A total of 2640 SSRs were identified across the 39 species, of which 2374 were A/T monomers, 57 were G/C monomers, and 209 were AT/TA/GA/TC dimers. No trimers, tetramers, hexamers, and pentamers were found. The number of tandem repeats was similar among the 39 species (4–9). However, the lengths of tandem repeats of *Cinnamomum* and *Sassafras* were 18–39 bp, smaller than *Nectandra* and *Ocotea* (19–99 bp).

### Phylogenetic analyses

**Fig. 3:**
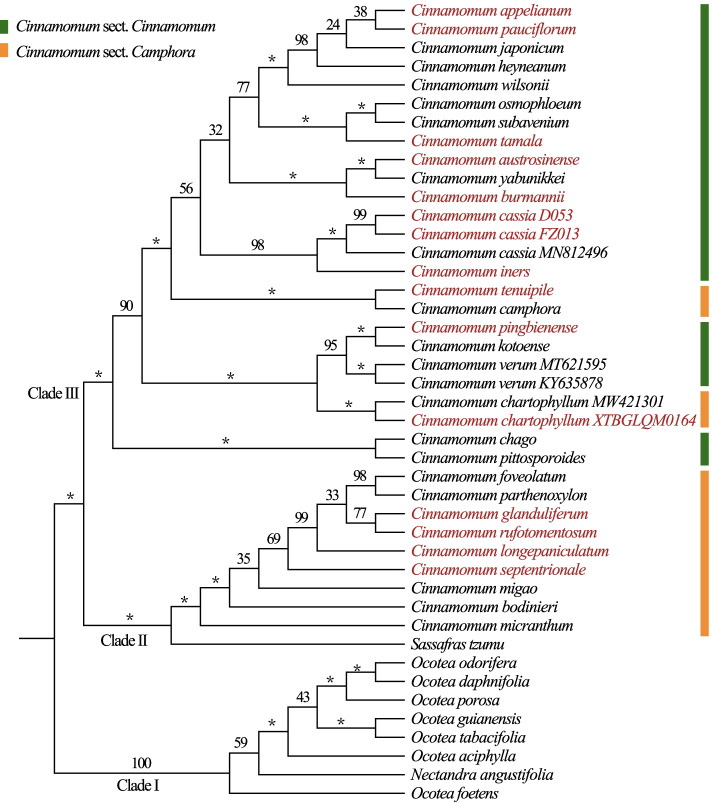
Phylogenetic tree inferred from maximum likelihood analysis based on concatenated protein-coding genes (PCG-c). Outgroups are pruned; bootstrap values = 100% are indicated as asterisks (*) above branches; newly sequenced samples are red-colored

The alignment lengths, number of variable sites, number of parsimony-informative sites, and GC content of PCG-c, NPCG-c, and CP-c are shown in Table 2. Because the phylogenetic relationships within tribe Cinnamomeae were largely congruent based on the three matrices (Figs. 3, S5, and S6), only the PCG-c ML tree has been present in the main text. As shown in Fig. 3, tribe Cinnamomeae consisted of three major clades—I, II, and III. *Nectandra* and *Ocotea* (clade I) were sister to *Sassafras* and *Cinnamomum* (clade II). In clade II, nine of the 12 species from *Cinnamomum* sect. *Camphora* formed a monophyletic group and were sister to *Sassafras*. In clade III, the other three species (*C. chartophyllum*, *C. camphora*, and *C. tenuipile*) of sect. *Camphora* were nested within 18 species from sect. *Cinnamomum*.

**Table 2:** Summary of the three matrices used in maximum likelihood analyses

Matrices	Alignment length (bp)	Number of variable sites	Number of parsimony informative sites	GC content (%)	Substitution models
CP-c	139,269	6114	2384	38.3	GTRGAMMA
NPCG-c	69,312	3560	1389	37.6	GTRGAMMA
PCG-c	69,957	2554	995	39.1	GTRGAMMA

### Selective pressure analyses

According to the site-specific model comparisons and LRT tests, 19 genes contained 57 positively selected sites. Of these genes, *ycf1* harbored 18 sites, with nine in *rbcL*, seven in *ycf2*, and 1–3 in each of the other 16 genes (*accD*, *ndhA*, *ndhF*, *ndhJ*, *petD*, *psaA*, *psbC*, *psaB*, *psbB*, *rpl2*, *rpl16*, *rpoC2*, *rpoB*, *rps12*, *rps2*, and *ycf4*; Table S7).

### Effect of uninformative loci on molecular dating

**Fig. 4:**
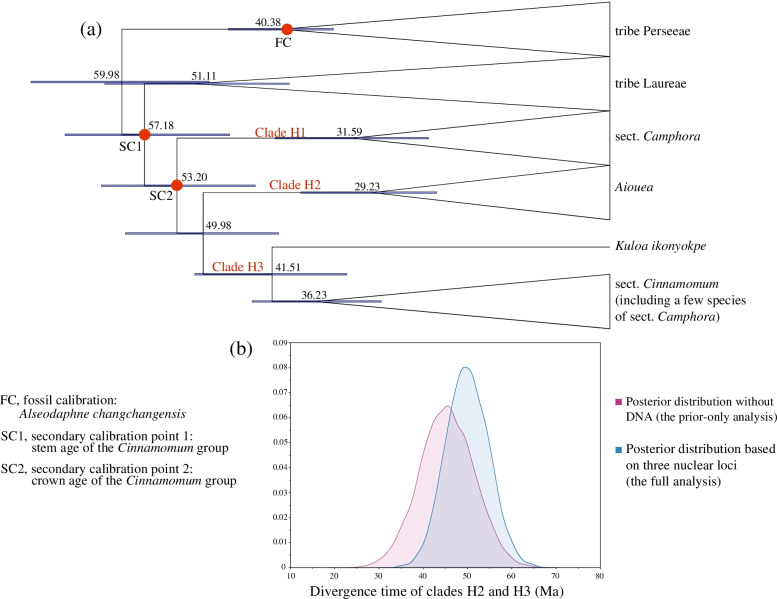
Divergence time estimation using ITS, *LEAFY*, and *RPB2*. **a** Molecular dating with DNA data (full analysis). The numbers near nodes are divergence times; the blue node bars indicate 95% highest posterior distributions; the three red circles at nodes indicate calibration points; species-rich clades are collapsed. **b** The posterior distributions of the divergence time of clades H1 and H2 in the full analysis and prior-only analysis (divergence time estimation without DNA data). Prior-only analysis and full analysis are colored in red and blue, respectively

According to BEAST analysis based on three nuclear loci (full analysis), clade H2 (*Aiouea*) separated from clade H3 (*Kuloa* + *C*. sect. *Cinnamomum*) at 49.98 Ma (95% highest posterior density (HPD) = 40.71–59.54 Ma) (Fig. 4a and b). BEAST analysis without DNA (prior-only analysis) showed that the divergence time of clades H2 and H3 was 45.35 Ma (95% HPD = 33.58–57.50 Ma) (Fig. 4b). The posterior distributions largely overlapped (Fig. 4b), and the means were similar (49.98 vs. 45.35), suggesting that the dating results of the full analysis were mainly determined by prior age constraints, rather than by the three nuclear loci data.

### Divergence times within tribe Cinnamomeae based on PCGs

**Fig. 5:**
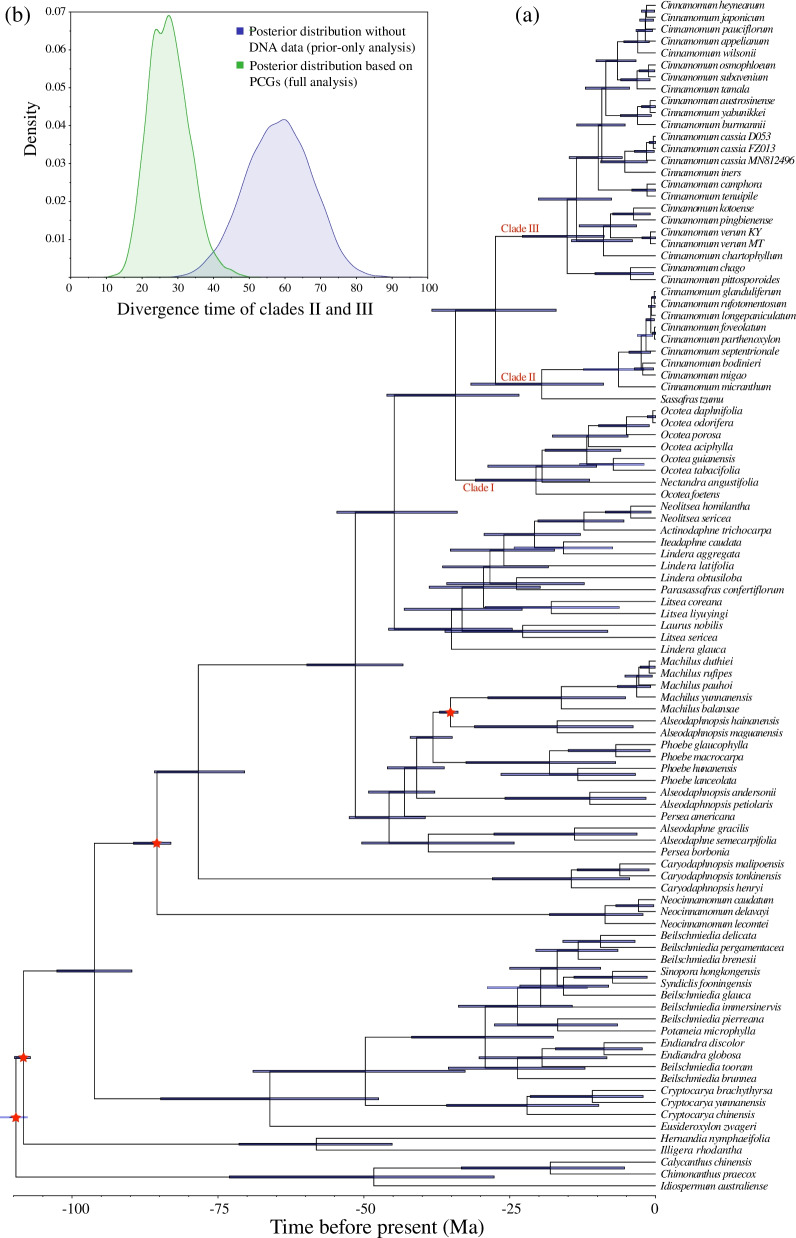
Divergence time estimation using plastid protein-coding genes (PCGs). **a** Molecular dating with DNA data (full analysis). The blue node bars indicate 95% highest posterior distributions; the four red pentacles indicate fossil calibration points. **b** The posterior distributions of the divergence time of clades II and III in the full analysis and prior-only analysis (divergence time estimation without DNA data). Prior-only analysis and full analysis are colored in blue and green, respectively

According to BEAST analysis based on PCGs (full analysis), tribe Cinnamomeae originated at 44.79 Ma (95% HPD = 34.02–54.64 Ma) and diverged at 34.31 Ma (95% HPD = 23.44–46.05 Ma) (Fig. 5a). Clade II separated from clade III at 27.47 Ma (95% HPD = 17.08–38.34 Ma) (Fig. 5b). BEAST analysis without PCGs (prior-only analysis) showed that the divergence time of clades II and III was 58.23 Ma (95% HPD = 39.81–75.16 Ma) (Fig. 5b). The posterior distributions did not overlap, and the means were substantially different (Fig. 5b), suggesting that the dating results of the full analysis were determined by PCGs, not by prior age constraints.

## Discussion

### Plastome structure variation and evolution

Fourteen of the 15 newly sequenced plastomes of *Cinnamomum* were conservative in overall structure, genome size, GC content, and gene order and content (Fig. 1; Tables 1 and S4), which were congruent with published plastomes from tribe Cinnamomeae [14, 60, 61]. One exception was the plastome of *Cinnamomum chartophyllum* XTBGLQM0164, which had a larger genome size compared with another published plastome of this species (MW421301, 152,722 bp) and the other 14 newly sequenced plastomes (Table 1; Fig. 1). Its larger size was caused by IR expansion, resulting in double complete *trnI*^*CAU*^, *rpl32*, *rpl2*, and *ycf2* in the IR regions (Figs. 1 and S1), which is the first case in tribe Cinnamomeae. Infrageneric IR expansion was relatively common in angiosperms, for example, IR of *Plantago* (Plantaginaceae) ranged from 24,955 bp to 38,644 bp [62], *Pelargonium* (Geraniaceae) from 38,036 bp to 87,724 bp [63], *Euphorbia* (Euphorbiaceae) from 26,434 bp to 43,573 bp [64], and *Caryodaphnopsis* (Lauraceae) from 20,036 bp to 25,601 bp [61]. As for intraspecific IR expansion, a double-strand break followed by strand invasion and recombination can result in intraspecific length polymorphism and was proposed to explain large and small IR expansions [65–67], which may be responsible for the IR expansion of *C. chartophyllum* XTBGLQM0164.

Abundant repetitive sequences were detected across the 39 species of tribe Cinnamomeae (Table S6). For SSRs, poly-A/T constituted the majority and poly-G/C were rare in this study, which were also found in other plants, such as *Euphorbia* [64], *Zygophyllum* [68], and *Swertia* [69]. Long repeat sequences play critical roles in plastome variation and rearrangements [65, 70]. Although abundant long repeats (dispersed and tandem repeats) were detected, no rearrangements were observed in the Mauve analysis (Fig. S3). Interestingly, the maximum lengths of long repeat sequences were substantially higher in *Nectandra* and *Ocotea* than in *Cinnamomum* and *Sassafras* (Table S6), which may reflect the distinct evolutionary histories of the two lineages of tribe Cinnamomeae. The newly identified SSRs, tandem repeats, and dispersed repeats can facilitate population genetics and evolutionary studies of tribe Cinnamomeae in the future.

Plastids are bioenergetic organelles responsible for photosynthesis and numerous metabolic processes. Positive selection of plastid genes is common and has been used to explain the adaptive evolution of plants [69, 71–73]. In this study, the site models indicated that positive selection acted on sites of roughly one-fifth of all plastid PCGs (19 of 79; Table S7). Of these genes, *ycf1*, *ycf2*, and *rbcL* contained more positively selected sites than the other genes. *ycf1* and *ycf2* are the two largest open reading frames of higher plants and encode products essential to cell survival [74]. *ycf2* was also reported to participate in encoding the 2-MD heteromeric AAA-ATPase complex, which associates with the TIC complex and functions as an import motor [75]. *rbcL* is a photosynthesis-related gene that encodes the large subunit of RubisCO and has been shown to undergo positive selection in all lineages of green plants [76]. For example, the positive selection in *rbcL* of *Schiedea* was suggested to promote the colonization of new habitats [77]. Therefore, the data generated in this study can facilitate future works that determine more specific details about how positive selection could have played a role in adaptations to new environments.

### Candidate DNA barcodes

DNA barcode is a standard region of nucleotide sequence used for species identification [78]. Three plastid loci (*rbcL*, *matK*, and *trnH*-*psbA*) and a nuclear-ribosomal DNA region (ITS2) were selected as standard barcodes [79] and were widely used in community ecology, biodiversity conservation, and evolutionary biology [80–82]. However, these standard barcodes always displayed low phylogenetic resolutions in recently diversified taxa [10, 83]; therefore, developing new DNA barcodes is necessary. This study showed that *ycf1*, *ycf2*, *petA*-*psbJ*, and *ndhF*-*rpl32*-*trnL*^*UAG*^ were more informative than the standard barcodes (*rbcL*, *matK*, and *trnH*-*psbA*), which were largely in line with Trofimov et al. [14]. *ycf1* was indicated to be the most variable loci and showed better phylogenetic resolutions than standard DNA barcodes in land plants [84]. *ycf2*, *petA*-*psbJ*, and *ndhF*-*rpl32*-*trnL*^*UAG*^ were not always hypervariable among different taxa [54, 64, 85], suggesting that the three loci were taxa-specific barcodes. Given the limited sampling in this study, more species with multiple samples of tribe Cinnamomeae should be included in future work to evaluate the discriminative power of *ycf2*, *petA*-*psbJ*, and *ndhF*-*rpl32*-*trnL*^*UAG*^.

### Phylogenetic relationships and divergence time of tribe Cinnamomeae

According to the PCG-c ML tree (Fig. 3), *Cinnamomum* and two of its sections were not monophyletic, which was consistent with Huang et al. [12]. *Cinnamomum camphora*, *C. chartophyllum*, and *C. tenuipile* were positioned in *C.* sect. *Camphora* based on ITS + *LEAFY* + *RPB2* [12], however, they were grouped with *C.* sect. *Cinnamomum* based on plastomes. The three species nested within different sections based on plastomes and nuclear loci, and originated long after the occurrence of the most recent common ancestor of *Cinnamomum*; therefore, their conflicting positions were unlikely to be caused by incomplete lineage sorting (ILS), which commonly occurred in a short period [86–88]. Thus, hybridization or introgression may be responsible for this case. Furthermore, the sister relationship of clades I and III was supported by ITS + *LEAFY* + *RPB2* [12]. In contrast, clade I was sister to clades II and III in this study. The contrasting cytonuclear discordance may be caused by ancient hybridization, introgression, or ILS, which are common in plants [16, 89, 90].

Divergence time estimation is the basis of historical biogeography, and inaccurate divergence time estimation can bias the understanding of plant evolution. By the full analysis and prior-only analysis comparison, the divergence times of tribe Cinnamomeae based on three nuclear loci were largely affected by prior age constraints (Fig. 4b) and thus were not accurate. Many branch support values of Huang et al. [12] were low, suggesting that the three nuclear loci had insufficient parsimony-informative sites and could have biased the molecular dating analysis [20]. In contrast, the PCGs results were not affected by the prior age constraints (Fig. 5b). According to the results, tribe Cinnamomeae originated around 44.79 Ma, about 10 Ma younger than the estimation from Huang et al. [12], and the divergence time of the two sections of *Cinnamomum* was 27.47 Ma, about 24 Ma younger than the estimation from Huang et al. [12]. Therefore, the biogeographic inference of Huang et al. [12] needs to be reinvestigated. For example, *Kuloa* is distributed in Central Africa and sister to *C.* sect. *Cinnamomum* [12, 91]. Its divergence from *C.* sect. *Cinnamomum* should be later than the divergence of *C.* sect. *Cinnamomum* and *C.* sect. *Camphora*, 27.47 Ma (Fig. 5a), which was long after the breakup of boreotropical flora in the late Eocene [92, 93]. Therefore, the Africa–Asia disjunction of tribe Cinnamomeae was more likely caused by long-distance dispersal rather than by the breakup of boreotropical flora. Despite the new findings in this study, more species and a large number of nuclear loci are needed to further elucidate the phylogenetic relationships and infer a more reasonable historical biogeography of tribe Cinnamomeae.

## Conclusions

In this study, 15 plastomes representing 14 species of tribe Cinnamomeae were newly sequenced. Comparative analyses showed that plastomes of tribe Cinnamomeae were highly similar in terms of the overall structure, long repeat sequences, and SSRs. Drastic expansion of the IR regions was detected in *Cinnamomum chartophyllum* XTBGLQM0164, which is the first case in tribe Cinnamomeae. *ycf1*, *ycf2*, *ndhF*-*rpl32*-*trnL*^*UAG*^, and *petA*-*psbJ* were hypervariable and can be used as candidate DNA barcodes for this tribe. Divergence time estimation using plastomes was not affected by prior age constraints. *Cinnamomum* sect. *Camphora* separated from *C.* sect. *Cinnamomum* at 27.47 Ma, long after the breakup of boreotropical flora, suggesting that long-distance dispersal may play an important role in shaping the disjunctive distribution of tribe Cinnamomeae. Overall, the obtained plastome resources can facilitate population genetics, phylogenetics, and biogeographic studies of tribe Cinnamomeae in the future.

## Supplementary Information

**Additional file 1: Table S1.** The plastomes used in different analyses of this study. **Table S2.** Collection information and accession numbers of the 15 samples of tribe Cinnamomeae. **Table S3.** The GenBank accession numbers of ITS, *RPB2*, and *LEAFY*. **Table S4.** Gene content of the 15 newly generated plastomes. **Table S5.** The sequences of primers. **Table S6.** Number of dispersed repeats, SSRs, and tandem repeats of the 39 species of tribe Cinnamomeae. **Table S7.***p* value of the likelihood ratio tests and positively selected codon sites.

**Additional file 2: Fig. S1.** Comparison of the SC/IR junctions among the 15 newly generated plastomes of tribe Cinnamomeae. JLA, LSC/IRa boundary; JSA, SSC/IRa boundary; JSB, SSC/IRb boundary; JLB, LSC/IRb boundary. **Fig. S2.** Map of the gel electrophoresis experiments. XTBG, *Cinnamomum chartophyllum*; D053, *C. cassia*; wh020, *C. longepaniculatum*. **Fig. S3.** Structural alignment of the 15 newly generated plastomes of tribe Cinnamomeae inferred from Mauve. **Fig. S4.** Visualized alignments of 39 plastomes of tribe Cinnamomeae using mVISTA. The vertical scale indicates percentage of identity ranging from 50 to 100%. Exons are colored in dark blue, non-coding sequences (CNS) are colored in red, tRNA and rRNA genes (UTR) are colored in green. **Fig. S5.** Phylogenetic tree inferred from maximum likelihood analysis using concatenated complete plastomes with one IR removed (CP-c). Bootstrap values are indicated above branches. **Fig. S6.** Phylogenetic tree inferred from maximum likelihood analysis using concatenated non-protein-coding genes (NPCG-c). Bootstrap values are indicated above branches.

## Data Availability

The newly sequenced 15 plastomes of tribe Cinnamomeae were submitted to the Science Data Bank (10.57760/sciencedb.01896) and GenBank (accession numbers shown in Table S2). All raw reads were submitted to sequence read archive of NCBI under bioproject PRJNA843587 (SRA accession numbers shown in Table S2). The accession numbers of the other plastomes downloaded from GenBank and LCGDB were shown in Table S1.

## Abbreviations

AIC: Akaike information criterion
BEB: Bayes empirical bayes
BGI: Beijing Genomics Institute
CP: Complete plastome
CTAB: Cetyltrimethylammonium bromide
ESS: Effective sample size
HPD: Highest posterior density
ILS: Incomplete lineage sorting
IR: Inverted repeat
LCGDB: Lauraceae Chloroplast Genome Database
LRT: Likelihood ratio test
LSC: Large single copy
MCMC: Markov chain Monte Carlo
ML: Maximum likelihood
NPCG: Non-protein-coding gene
OGDRAW: OrganellarGenomeDRAW
PCG: Protein-coding gene
PCR: Polymerase chain reaction
Pi: Parsimony-informative
RAD-seq: Restriction site-associated DNA sequencing
SSC: Small single copy
SSR: Simple sequence repeat
