# Supplementary material for: Plastome structure, phylogenomics, and divergence times of tribe Cinnamomeae (Lauraceae)
Source: BMC Genomics. 2022 Sep 8;23:642. doi: 10.1186/s12864-022-08855-4 (PMC9461114; doi:10.1186/s12864-022-08855-4)
Supplement: Supplementary file 1 — Additional file 1: Table S1. The plastomes used in different analyses of this study. Table S2. Collection information and accession numbers of the 15 samples of tribe Cinnamomeae. Table S3. The GenBank accession numbers of ITS, RPB2, and LEAFY. Table S4. Gene content of the 15 newly generated plastomes. Table S5. The sequences of primers. Table S6. Number of dispersed repeats, SSRs, and tandem repeats of the 39 species of tribe Cinnamomeae. Table S7.p value of the likelihood ratio tests and positively selected codon sites. [file 12864_2022_8855_MOESM1_ESM.zip › Additional file 1 Table S1.docx]

**Additional file 1: Table S1.** The plastomes used in different analyses of this study.

| **Taxon** | **Accession No. / Voucher No.** | **Used in IRscope and Mauve** | **Used in mVISTA, DnaSP and repeat sequence analyses** | **Used in phylogenetic and selective pressure analyses** | **Used in BEAST 2 analyses** |
| --- | --- | --- | --- | --- | --- |
| *Actinodaphne trichocarpa* | MF939342 | - | - | - | Yes |
| *Alseodaphne gracilis* | MG407593 | - | - | - | Yes |
| *Alseodaphne semecarpifolia* | MG407595 | - | - | - | Yes |
| *Alseodaphnopsis andersonii* | MT621619 | - | - | - | Yes |
| *Alseodaphnopsis hainanensis* | LAU00036 | - | - | - | Yes |
| *Alseodaphnopsis maguanensis* | MT621609 | - | - | - | Yes |
| *Alseodaphnopsis petiolaris* | MT621634 | - | - | - | Yes |
| *Beilschmiedia brenesii* | MT720933 | - | - | - | Yes |
| *Beilschmiedia brunnea* | MT720935 | - | - | - | Yes |
| *Beilschmiedia delicata* | MT720936 | - | - | - | Yes |
| *Beilschmiedia glauca* | MT720938 | - | - | - | Yes |
| *Beilschmiedia immersinervis* | MT720939 | - | - | - | Yes |
| *Beilschmiedia pergamentacea* | MT720941 | - | - | - | Yes |
| *Beilschmiedia pierreana* | MT720942 | - | - | - | Yes |
| *Beilschmiedia tooram* | MT720944 | - | - | - | Yes |
| *Calycanthus chinensis* | MH377059 | - | - | - | Yes |
| *Caryodaphnopsis henryi* | MF939346 | - | - | - | Yes |
| *Caryodaphnopsis malipoensis* | MT621621 | - | - | - | Yes |
| *Caryodaphnopsis tonkinensis* | MT621583 | - | - | - | Yes |
| *Chimonanthus praecox* | NC042744 | - | - | - | Yes |
| ***Cinnamomum appelianum*** | CFL3846 | Yes | Yes | Yes | Yes |
| ***Cinnamomum austrosinense*** | 2520043 | Yes | Yes | Yes | Yes |
| *Cinnamomum bodinieri* | MH394415 | - | Yes | Yes | Yes |
| ***Cinnamomum burmannii*** | XTBGLQM0487 | Yes | Yes | Yes | Yes |
| *Cinnamomum camphora* | MT621650 | - | Yes | Yes | Yes |
| ***Cinnamomum cassia*** | D053 | Yes | Yes | Yes | Yes |
| ***Cinnamomum cassia*** | FZ013 | Yes | - | Yes | Yes |
| *Cinnamomum cassia* | MN812496 | - | Yes | Yes | Yes |
| *Cinnamomum chago* | LAU00078 | - | Yes | Yes | Yes |
| ***Cinnamomum chartophyllum*** | XTBGLQM0164 | Yes | Yes | Yes | Yes |
| *Cinnamomum chartophyllum* | MW421301 | - | - | Yes | - |
| *Cinnamomum foveolatum* | MT621633 | - | Yes | Yes | Yes |
| ***Cinnamomum glanduliferum*** | CFL2920 | Yes | Yes | Yes | Yes |
| *Cinnamomum heyneanum* | LAU00047 | - | Yes | Yes | Yes |
| ***Cinnamomum iners*** | XTBGLQM0484 | Yes | Yes | Yes | Yes |
| *Cinnamomum japonicum* | MT621639 | - | Yes | Yes | Yes |
| *Cinnamomum kotoense* | MN698964 | - | Yes | Yes | Yes |
| ***Cinnamomum longepaniculatum*** | wh020 | Yes | Yes | Yes | Yes |
| *Cinnamomum micranthum* | KT833081 | - | Yes | Yes | Yes |
| *Cinnamomum migao* | MZ433384 | - | Yes | Yes | Yes |
| *Cinnamomum osmophloeum* | MT384386 | - | Yes | Yes | Yes |
| *Cinnamomum parthenoxylon* | MT621587 | - | Yes | Yes | Yes |
| ***Cinnamomum pauciflorum*** | CFL3983 | Yes | Yes | Yes | Yes |
| ***Cinnamomum pingbienense*** | XTBGLQM0740 | Yes | Yes | Yes | Yes |
| *Cinnamomum pittosporoides* | MW801188 | - | Yes | Yes | Yes |
| ***Cinnamomum rufotomentosum*** | CFL2798 | Yes | Yes | Yes | Yes |
| ***Cinnamomum septentrionale*** | HZ105 | Yes | Yes | Yes | Yes |
| *Cinnamomum subavenium* | MW801140 | - | Yes | Yes | Yes |
| ***Cinnamomum tamala*** | XTBGLQM0255 | Yes | Yes | Yes | Yes |
| ***Cinnamomum tenuipile*** | XTBGLQM0666 | Yes | Yes | Yes | Yes |
| *Cinnamomum verum* | KY635878 | - | - | Yes | Yes |
| *Cinnamomum verum* | MT621595 | - | Yes | Yes | Yes |
| *Cinnamomum wilsonii* | MW800949 | - | Yes | Yes | Yes |
| *Cinnamomum yabunikkei* | MG717939 | - | Yes | Yes | Yes |
| *Cryptocarya brachythyrsa* | MT621601 | - | - | - | Yes |
| *Cryptocarya chinensis* | MF939349 | - | - | - | Yes |
| *Cryptocarya yunnanensis* | MT621600 | - | - | - | Yes |
| *Endiandra discolor* | KT588615 | - | - | - | Yes |
| *Endiandra globosa* | KT588614 | - | - | - | Yes |
| *Eusideroxylon zwageri* | MF939351 | - | - | - | Yes |
| *Hernandia nymphaeifolia* | MG838431 | - | - | - | Yes |
| *Idiospermum australiense* | MH377056 | - | - | - | Yes |
| *Illigera rhodantha* | MW423610 | - | - | - | Yes |
| *Iteadaphne caudata* | LAU00055 | - | - | Yes | Yes |
| *Laurus nobilis* | KY085912 | - | - | Yes | Yes |
| *Lindera aggregata* | MG581437 | - | - | Yes | Yes |
| *Lindera glauca* | MH220732 | - | - | Yes | Yes |
| *Lindera latifolia* | MH220733 | - | - | Yes | Yes |
| *Lindera obtusiloba* | MH220737 | - | - | Yes | Yes |
| *Litsea coreana* | MG581436 | - | - | Yes | Yes |
| *Litsea liyuyingi* | MT621575 | - | - | Yes | Yes |
| *Litsea sericea* | MT621622 | - | - | Yes | Yes |
| *Machilus balansae* | KT348517 | - | - | - | Yes |
| *Machilus duthiei* | MT621635 | - | - | - | Yes |
| *Machilus pauhoi* | MH178403 | - | - | - | Yes |
| *Machilus rufipes* | MT621629 | - | - | - | Yes |
| *Machilus yunnanensis* | KT348516 | - | - | - | Yes |
| *Nectandra angustifolia* | MF939340 | - | Yes | Yes | Yes |
| *Neocinnamomum caudatum* | MT621645 | - | - | - | Yes |
| *Neocinnamomum delavayi* | LC213014 | - | - | - | Yes |
| *Neocinnamomum lecomtei* | MF939345 | - | - | - | Yes |
| *Neolitsea homilantha* | MT621638 | - | - | Yes | Yes |
| *Neolitsea sericea* | MF939341 | - | - | Yes | Yes |
| *Ocotea aciphylla* | OM135246 | - | Yes | Yes | Yes |
| *Ocotea daphnifolia* | OM135247 | - | Yes | Yes | Yes |
| *Ocotea foetens* | OM135248 | - | Yes | Yes | Yes |
| *Ocotea guianensis* | OM135249 | - | Yes | Yes | Yes |
| *Ocotea odorifera* | OM135250 | - | Yes | Yes | Yes |
| *Ocotea porosa* | OM135251 | - | Yes | Yes | Yes |
| *Ocotea tabacifolia* | OM135252 | - | Yes | Yes | Yes |
| *Parasassafras confertiflorum* | MH729378 | - | - | Yes | Yes |
| *Persea americana* | KX437771 | - | - | - | Yes |
| *Persea borbonia* | LAU00074 | - | - | - | Yes |
| *Phoebe glaucophylla* | MZ433408 | - | - | - | Yes |
| *Phoebe hunanensis* | MZ433409 | - | - | - | Yes |
| *Phoebe lanceolata* | MZ433411 | - | - | - | Yes |
| *Phoebe macrocarpa* | MZ433412 | - | - | - | Yes |
| *Potameia microphylla* | MT720950 | - | - | - | Yes |
| *Sassafras tzumu* | MW800928 | - | Yes | Yes | Yes |
| *Sinopora hongkongensis* | MN722652 | - | - | - | Yes |
| *Syndiclis fooningensis* | MT720952 | - | - | - | Yes |

**Note:** Taxa in bold indicate newly sequenced species/samples in this study.
