# Supplementary material for: Plastome structure, phylogenomics, and divergence times of tribe Cinnamomeae (Lauraceae)
Source: BMC Genomics. 2022 Sep 8;23:642. doi: 10.1186/s12864-022-08855-4 (PMC9461114; doi:10.1186/s12864-022-08855-4)
Supplement: Supplementary file 1 — Additional file 1: Table S1. The plastomes used in different analyses of this study. Table S2. Collection information and accession numbers of the 15 samples of tribe Cinnamomeae. Table S3. The GenBank accession numbers of ITS, RPB2, and LEAFY. Table S4. Gene content of the 15 newly generated plastomes. Table S5. The sequences of primers. Table S6. Number of dispersed repeats, SSRs, and tandem repeats of the 39 species of tribe Cinnamomeae. Table S7.p value of the likelihood ratio tests and positively selected codon sites. [file 12864_2022_8855_MOESM1_ESM.zip › Additional file 1 Table S3.docx]

**Additional file 1: Table S3.** The GenBank accession numbers of ITS, *RPB2*, and *LEAFY*.

| **Taxon** | **ITS** | **RPB2** | **LEAFY** |
| --- | --- | --- | --- |
| *Aiouea dubia* | AF272250 | – | – |
| *Aiouea dubia* | KU139905 | – | – |
| *Aiouea guianensis* | AF272251 | – | – |
| *Alseodaphne hainanensis* | FJ755440 | KU140409 | HQ697006 |
| *Alseodaphne rugosa* | HQ6971813 | KU140410 | HQ697011 |
| *Cinnamomum appelianum* | KU139817 | KU140330 | KU140244 |
| *Cinnamomum austrosinense* | KU139818 | KU140331 | KU140245 |
| *Cinnamomum austroyunnanense* | KU139819 | KU140332 | KU140246 |
| *Cinnamomum baileyanum* | KU139820 | KU140333 | KU140247 |
| *Cinnamomum baileyanum* | KU139821 | KU140334 | KU140248 |
| *Cinnamomum bejolghota* | KU139822 | KU140335 | KU140249 |
| *Cinnamomum bejolghota* | KU139823 | – | KU140250 |
| *Cinnamomum bodinieri* | KU139824 | KU140336 | KU140251 |
| *Cinnamomum burmannii* | KU139825 | KU140337 | KU140252 |
| *Cinnamomum camphora* | KU139826 | KU140338 | KU140253 |
| *Cinnamomum cassia* | KU139827 | KU140339 | KU140254 |
| *Cinnamomum cassia* | KU139828 | KU140340 | KU140255 |
| *Cinnamomum celebicum* | KU139829 | KU140341 | KU140256 |
| *Cinnamomum chaogo* | KU139830 | KU140342 | KU140257 |
| *Cinnamomum chartophyllum* | KU139832 | KU140344 | KU140259 |
| *Cinnamomum chartophyllum* | KU139831 | KU140343 | KU140258 |
| *Cinnamomum chavarrinum* | KU139833 | KU140345 | – |
| *Cinnamomum chekiangense* | KU139834 | KU140346 | KU140260 |
| *Cinnamomum cinnamomifolium* | AF272262 | – | – |
| *Cinnamomum cordatum* | KU139835 | KU140347 | KU140261 |
| *Cinnamomum costaricanum* | KU139836 | KU140348 | KU140262 |
| *Cinnamomum costaricanum* | KU139837 | KU140349 | KU140263 |
| *Cinnamomum crenulicupulum* | KU139838 | KU140350 | KU140264 |
| *Cinnamomum cuspidatum* | KU139839 | KU140351 | KU140265 |
| *Cinnamomum daphnoides* | KU139841 | KU140353 | KU140267 |
| *Cinnamomum daphnoides* | KU139840 | KU140352 | KU140266 |
| *Cinnamomum doederleinii* | KU139842 | – | KU140268 |
| *Cinnamomum glanduliferum* | KU139843 | KU140354 | KU140269 |
| *Cinnamomum grandifolium* | KU139844 | KU140355 | KU140270 |
| *Cinnamomum haussknechtii* | KU139845 | KU140356 | KU140271 |
| *Cinnamomum haussknechtii* | KU139846 | KU140357 | KU140272 |
| *Cinnamomum heyneanum* | KU139847 | KU140358 | KU140273 |
| *Cinnamomum iners* | KU139848 | KU140359 | KU140274 |
| *Cinnamomum iners* | KU139849 | KU140360 | KU140275 |
| *Cinnamomum insularimontanum* | KU139850 | – | KU140276 |
| *Cinnamomum japonicum* | KU139851 | KU140361 | KU140277 |
| *Cinnamomum javanicum* | KU139852 | KU140362 | KU140278 |
| *Cinnamomum jensenianum* | KU139853 | KU140363 | KU140279 |
| *Cinnamomum kotoense* | KU139854 | KU140364 | KU140280 |
| *Cinnamomum laubatii* | KU139855 | KU140365 | KU140281 |
| *Cinnamomum liangii* | KU139856 | KU140366 | KU140282 |
| *Cinnamomum longepaniculatum* | KU139857 | KU140367 | KU140283 |
| *Cinnamomum longipetiolatum* | KU139858 | – | KU140284 |
| *Cinnamomum mairei* | KU139859 | KU140368 | KU140285 |
| *Cinnamomum micranthum* | KU139860 | KU140369 | KU140286 |
| *Cinnamomum mollifolium* | KU139861 | KU140370 | KU140287 |
| *Cinnamomum multiflorum* | KU139862 | KU140371 | KU140288 |
| *Cinnamomum okinawense* | KU139863 | – | KU140289 |
| *Cinnamomum oleifolium* | KU139864 | KU140372 | KU140290 |
| *Cinnamomum oliveri* | KU139865 | KU140373 | KU140291 |
| *Cinnamomum osmophloeum* | KU139867 | KU140375 | – |
| *Cinnamomum osmophloeum* | KU139866 | KU140374 | – |
| *Cinnamomum padiforme* | KU139868 | – | KU140292 |
| *Cinnamomum parthenoxylon* | KU139869 | KU140376 | KU140293 |
| *Cinnamomum parthenoxylon* | KU139870 | – | KU140294 |
| *Cinnamomum parthenoxylon* | KU139871 | KU140377 | KU140295 |
| *Cinnamomum pauciflorum* | KU139872 | KU140378 | KU140296 |
| *Cinnamomum pingbienense* | KU139873 | KU140379 | KU140297 |
| *Cinnamomum pittosporoides* | KU139874 | KU140380 | KU140298 |
| *Cinnamomum platyphyllum* | KU139875 | KU140381 | KU140299 |
| *Cinnamomum propinquum* | KU139877 | KU140383 | KU140301 |
| *Cinnamomum propinquum* | KU139876 | KU140382 | KU140300 |
| *Cinnamomum quadrangulum* | KU139878 | KU140384 | KU140302 |
| *Cinnamomum reticulatum* | KU139879 | – | KU140303 |
| *Cinnamomum rhynchophyllum* | KU139880 | KU140385 | KU140304 |
| *Cinnamomum rigidissimum* | KU139881 | KU140386 | KU140305 |
| *Cinnamomum saxatile* | KU139882 | KU140387 | KU140306 |
| *Cinnamomum septentrionale* | KU139883 | KU140388 | KU140307 |
| *Cinnamomum sintoc* | KU139884 | KU140389 | KU140308 |
| *Cinnamomum* sp. | KU139886 | KU140391 | KU140310 |
| *Cinnamomum* sp. | KU139885 | KU140390 | KU140309 |
| *Cinnamomum* sp. | KU139887 | KU140392 | KU140311 |
| *Cinnamomum subavenium* | KU139888 | KU140393 | KU140312 |
| *Cinnamomum subsessile* | KU139889 | KU140394 | KU140313 |
| *Cinnamomum subsessile* | KU139890 | KU140395 | KU140314 |
| *Cinnamomum tamala* | KU139891 | KU140396 | KU140315 |
| *Cinnamomum tenuifolium* | KU139892 | KU140397 | KU140316 |
| *Cinnamomum tenuipile* | KU139893 | KU140398 | KU140317 |
| *Cinnamomum tomentulosum* | KU139894 | KU140399 | KU140318 |
| *Cinnamomum tonkinense* | KU139895 | KU140400 | KU140319 |
| *Cinnamomum tonkinense* | KU139896 | KU140401 | KU140320 |
| *Cinnamomum triplinerve* | KU139897 | KU140402 | KU140321 |
| *Cinnamomum triplinerve* | KU139898 | KU140403 | KU140322 |
| *Cinnamomum triplinerve* | KU139899 | KU140404 | KU140323 |
| *Cinnamomum tsangii* | KU139900 | KU140405 | KU140324 |
| *Cinnamomum tsoi* | KU139901 | KU140406 | KU140325 |
| *Cinnamomum verum* | KU139902 | KU140407 | KU140326 |
| *Cinnamomum verum* | KU139903 | – | KU140327 |
| *Cinnamomum wilsonii* | KU139904 | KU140408 | KU140328 |
| *Laurus nobilis* | KU139906 | KU140411 | KU140329 |
| *Lindera erythrocarpa* | HQ697215 | KU140412 | HQ697170 |
| *Lindera megaphylla* | HQ697216 | – | HQ697173 |
| *Litsea auriculata* | HQ697217 | – | HQ697174 |
| *Litsea verticillata* | HQ697218 | – | HQ697175 |
| *Mocinnodaphne cinnamomoidea* | AF272288 | – | – |
| *Neolitsea cambodiana* | HQ697219 | – | HQ697176 |
| *Neolitsea howii* | HQ697220 | – | HQ697178 |
| *Neolitsea sericea* | HQ697221 | KT248756 | HQ697180 |
| *Ocotea ikonyokpe* | AF272305 | – | – |
| *Phoebe hungmoensis* | HQ697206 | KU140413 | HQ697138 |
| *Phoebe nanmu* | FJ755409 | KT248757 | HQ697149 |
| *Phoebe zhennan* | HQ697212 | KT248761 | HQ697161 |
| *Phoebe glaucifolia* | – | KT248758 | – |

**Note:** "–" indicate missing data.
