# Supplementary material for: Plastome structure, phylogenomics, and divergence times of tribe Cinnamomeae (Lauraceae)
Source: BMC Genomics. 2022 Sep 8;23:642. doi: 10.1186/s12864-022-08855-4 (PMC9461114; doi:10.1186/s12864-022-08855-4)
Supplement: Supplementary file 1 — Additional file 1: Table S1. The plastomes used in different analyses of this study. Table S2. Collection information and accession numbers of the 15 samples of tribe Cinnamomeae. Table S3. The GenBank accession numbers of ITS, RPB2, and LEAFY. Table S4. Gene content of the 15 newly generated plastomes. Table S5. The sequences of primers. Table S6. Number of dispersed repeats, SSRs, and tandem repeats of the 39 species of tribe Cinnamomeae. Table S7.p value of the likelihood ratio tests and positively selected codon sites. [file 12864_2022_8855_MOESM1_ESM.zip › Additional file 1 Table S6.docx]

**Additional file 1: Table S6.** Number of dispersed repeats, SSRs, and tandem repeats of the 39 species of tribe Cinnamomeae.

| **Taxa** | **Dispersed repeats** | | | | | | **SSRs** | | | | **Tandem repeats** | |
| --- | --- | --- | --- | --- | --- | --- | --- | --- | --- | --- | --- | --- |
|  | **Forward** | **Palindromic** | **Reverse** | **Complement** | **Length bp** | **A/T** | **G/C** | **AT/TA** | **GA** | **TC** | **Number** | **Length bp** |
| **Cinnamomum appelianum CFL3846** | 20 | 16 | 13 | 1 | 18–54 | 62 | 1 | 3 | 1 | 2 | 6 | 18–36 |
| **Cinnamomum austrosinense 2520043** | 21 | 10 | 17 | 2 | 19–51 | 61 | 4 | 3 | 1 | 2 | 5 | 18–36 |
| Cinnamomum bodinieri MH394415 | 17 | 12 | 19 | 2 | 19–57 | 68 | 0 | 3 | 0 | 2 | 7 | 19–36 |
| **Cinnamomum burmannii XTBGLQM0487** | 23 | 16 | 10 | 1 | 18–51 | 63 | 3 | 3 | 1 | 2 | 5 | 18–36 |
| Cinnamomum camphora MT621650 | 20 | 13 | 15 | 2 | 18–51 | 59 | 2 | 3 | 1 | 2 | 5 | 18–36 |
| **Cinnamomum cassia D053** | 16 | 13 | 16 | 5 | 18–48 | 57 | 0 | 3 | 1 | 2 | 3 | 19–24 |
| Cinnamomum chago LAU00078 | 22 | 14 | 13 | 1 | 18–66 | 59 | 1 | 3 | 1 | 2 | 5 | 18–36 |
| **Cinnamomum chartophyllum XTBGLQM0164** | 17 | 16 | 16 | 1 | 18–55 | 59 | 2 | 3 | 1 | 2 | 4 | 19–36 |
| Cinnamomum foveolatum MT621633 | 15 | 16 | 14 | 5 | 19–48 | 67 | 0 | 3 | 0 | 2 | 7 | 18–36 |
| **Cinnamomum glanduliferum CFL2920** | 14 | 18 | 12 | 6 | 18–48 | 67 | 0 | 3 | 0 | 2 | 8 | 18–36 |
| Cinnamomum heyneanum LAU00047 | 21 | 12 | 14 | 3 | 18–51 | 63 | 1 | 3 | 1 | 2 | 5 | 18–36 |
| **Cinnamomum iners XTBGLQM0484** | 23 | 13 | 12 | 2 | 18–49 | 58 | 2 | 3 | 1 | 2 | 5 | 18–36 |
| Cinnamomum japonicum MT621639 | 19 | 14 | 17 | 0 | 18–51 | 61 | 1 | 3 | 1 | 2 | 5 | 18–36 |
| Cinnamomum kotoense MN698964 | 17 | 14 | 17 | 2 | 18–51 | 54 | 1 | 2 | 1 | 2 | 5 | 18–36 |
| **Cinnamomum longepaniculatum wh020** | 15 | 20 | 12 | 3 | 19–54 | 67 | 0 | 3 | 0 | 2 | 8 | 18–36 |
| Cinnamomum micranthum KT833081 | 17 | 16 | 14 | 3 | 19–48 | 65 | 2 | 3 | 0 | 2 | 6 | 18–36 |
| Cinnamomum migao MZ433384 | 14 | 18 | 15 | 3 | 19–48 | 66 | 0 | 3 | 0 | 2 | 9 | 18–36 |
| Cinnamomum osmophloeum MT384386 | 24 | 12 | 12 | 2 | 18–66 | 59 | 2 | 2 | 1 | 2 | 5 | 18–36 |
| Cinnamomum parthenoxylon MT621587 | 17 | 16 | 13 | 4 | 19–71 | 66 | 0 | 3 | 0 | 2 | 7 | 18–36 |
| **Cinnamomum pauciflorum CFL3983** | 22 | 13 | 13 | 2 | 18–51 | 60 | 2 | 3 | 1 | 2 | 5 | 18–36 |
| **Cinnamomum pingbienense XTBGLQM0740** | 21 | 15 | 14 | 0 | 18–51 | 54 | 2 | 2 | 1 | 2 | 4 | 19–39 |
| Cinnamomum pittosporoides MW801188 | 19 | 16 | 12 | 3 | 18–53 | 60 | 2 | 3 | 1 | 1 | 5 | 18–36 |
| **Cinnamomum rufotomentosum CFL2798** | 15 | 19 | 12 | 4 | 19–54 | 67 | 0 | 3 | 0 | 2 | 8 | 18–36 |
| **Cinnamomum septentrionale HZ105** | 17 | 13 | 15 | 5 | 19–48 | 68 | 0 | 3 | 0 | 2 | 9 | 18–36 |
| Cinnamomum subavenium MW801140 | 22 | 15 | 11 | 2 | 18–87 | 58 | 2 | 2 | 1 | 2 | 5 | 18–36 |
| **Cinnamomum tamala XTBGLQM0255** | 22 | 13 | 12 | 3 | 18–51 | 58 | 0 | 2 | 1 | 2 | 5 | 18–36 |
| **Cinnamomum tenuipile XTBGLQM0666** | 20 | 14 | 16 | 0 | 18–54 | 60 | 1 | 3 | 1 | 2 | 5 | 18–36 |
| Cinnamomum verum MT621595 | 21 | 13 | 15 | 1 | 18–51 | 58 | 2 | 3 | 1 | 2 | 6 | 18–36 |
| Cinnamomum wilsonii MW800949 | 18 | 16 | 15 | 1 | 18–54 | 62 | 2 | 3 | 1 | 2 | 6 | 18–36 |
| Cinnamomum yabunikkei MG717939 | 18 | 11 | 21 | 0 | 19–51 | 61 | 3 | 3 | 1 | 2 | 5 | 18–36 |
| Nectandra angustifolia MF939340 | 22 | 9 | 19 | 0 | 19–275 | 58 | 2 | 3 | 1 | 1 | 6 | 19–61 |
| Ocotea aciphylla OM135246 | 19 | 13 | 17 | 1 | 18–99 | 57 | 3 | 2 | 1 | 2 | 7 | 19–98 |
| Ocotea daphnifolia OM135247 | 15 | 14 | 15 | 6 | 18–98 | 55 | 1 | 2 | 1 | 2 | 7 | 18–98 |
| Ocotea foetens OM135248 | 18 | 12 | 19 | 1 | 18–66 | 60 | 2 | 1 | 1 | 2 | 5 | 19–63 |
| Ocotea guianensis OM135249 | 17 | 15 | 18 | 0 | 19–100 | 61 | 4 | 2 | 1 | 2 | 7 | 19–98 |
| Ocotea odorifera OM135250 | 9 | 17 | 16 | 8 | 35–100 | 57 | 0 | 2 | 1 | 2 | 6 | 19–98 |
| Ocotea porosa OM135251 | 18 | 15 | 14 | 3 | 18–99 | 58 | 2 | 2 | 1 | 2 | 6 | 19–99 |
| Ocotea tabacifolia OM135252 | 12 | 18 | 15 | 5 | 19–100 | 62 | 4 | 2 | 1 | 2 | 6 | 19–98 |
| Sassafras tzumu MW800928 | 19 | 18 | 12 | 1 | 18–48 | 59 | 1 | 2 | 1 | 2 | 5 | 18–24 |

**Note:** Taxa names in bold indicate newly sequenced species in this study.
