# Supplementary material for: Plastome structure, phylogenomics, and divergence times of tribe Cinnamomeae (Lauraceae)
Source: BMC Genomics. 2022 Sep 8;23:642. doi: 10.1186/s12864-022-08855-4 (PMC9461114; doi:10.1186/s12864-022-08855-4)
Supplement: Supplementary file 1 — Additional file 1: Table S1. The plastomes used in different analyses of this study. Table S2. Collection information and accession numbers of the 15 samples of tribe Cinnamomeae. Table S3. The GenBank accession numbers of ITS, RPB2, and LEAFY. Table S4. Gene content of the 15 newly generated plastomes. Table S5. The sequences of primers. Table S6. Number of dispersed repeats, SSRs, and tandem repeats of the 39 species of tribe Cinnamomeae. Table S7.p value of the likelihood ratio tests and positively selected codon sites. [file 12864_2022_8855_MOESM1_ESM.zip › Additional file 1 Table S7.docx]

**Additional file 1: Table S7.** *p* value of the likelihood ratio tests and positively selected codon sites.

| **Gene** | ***p*_M2vs.M0** | ***p*_M2avs.M1a** | ***p*_M8vs.M7** | **Positively selected sites** |
| --- | --- | --- | --- | --- |
| *accD* | 1.18E-07 | 1.53E-05 | 1.23E-05 | 174P |
| *atpA* | 0.33162602 | 0.673924629 | 0.638366314 |  |
| *atpB* | 0.04634592 | 0.081136042 | 0.076882961 |  |
| *atpE* | 1 | 0.999895006 | 1 |  |
| *atpF* | 1 | 0.999976 | 0.261073057 |  |
| *atpH* | 0.99999941 | 0.999899005 | 0.999900005 |  |
| *atpI* | 1 | 0.999957001 | 0.999742033 |  |
| *ccsA* | 0.03627859 | 0.087699244 | 0.083457814 |  |
| *cemA* | 0.89677277 | 0.838513163 | 0.834647332 |  |
| *clpP* | 0.03122158 | 0.081160062 | 0.025579271 |  |
| *infA* | 1 | 0.999968001 | 0.922651213 |  |
| *matK* | 0.03357386 | 0.183813431 | 0.156952197 |  |
| *ndhA* | 3.22E-07 | 0.001208926 | 0.000618793 | 24Y,282F,288F |
| *ndhB* | 0.99999996 | 0.695516878 | 0.695523138 |  |
| *ndhC* | 0.0568973 | 0.019167349 | 0.0346893 |  |
| *ndhD* | 0.91935512 | 1 | 0.999674053 |  |
| *ndhE* | 0.26473679 | 0.355120894 | 0.209042646 |  |
| *ndhF* | 5.35E-12 | 0.000167458 | 0.000112116 | 587Q,653V |
| *ndhG* | 0.03282483 | 0.200763637 | 0.184624361 |  |
| *ndhH* | 0.94608634 | 0.892886327 | 0.872813829 |  |
| *ndhI* | 0.41101893 | 0.768766497 | 0.712508811 |  |
| *ndhJ* | 4.01E-05 | 0.000225476 | 0.000348325 | 5L |
| *ndhK* | 1 | 0.999986 | 0.997352511 |  |
| *petA* | 1 | 0.999987 | 0.814732859 |  |
| *petB* | 1 | 0.99999 | 0.99999 |  |
| *petD* | 0.00048184 | 0.021052731 | 0.01926385 | 5V |
| *petG* | 0.99999998 | 0.999973 | 0.999935002 |  |
| *petL* | 0.99999982 | 0.999924003 | 0.9995521 |  |
| *petN* | 0.99865118 | 0.985097163 | 0.977857818 |  |
| *psaA* | 1.87E-09 | 3.92E-05 | 4.39E-05 | 152N,258S |
| *psaB* | 0.00022407 | 0.045893307 | 0.028915948 | 659I |
| *psaC* | 0.99999737 | 0.999803019 | 0.005211029 |  |
| *psaI* | 1 | 0.999992 | 0.999947001 |  |
| *psaJ* | 0.99999998 | 0.999972 | 0.999789022 |  |
| *psbA* | 8.38E-05 | 0.038641981 | 0.006964984 |  |
| *psbB* | 1.43E-08 | 7.66E-05 | 7.05E-06 | 494A |
| *psbC* | 0.00124251 | 0.011522991 | 0.016475629 | 284V |
| *psbD* | 1 | 0.999960001 | 0.999961001 |  |
| *psbE* | 0.99999808 | 0.999828015 | 0.999996 |  |
| *psbF* | 1 | 0.999983 | 0.999999 |  |
| *psbH* | 0.22399226 | 0.475594347 | 0.452152847 |  |
| *psbI* | 0.99999984 | 0.999961001 | 0.999962001 |  |
| *psbJ* | 0.19040867 | 0.435314727 | 0.416914965 |  |
| *psbK* | 1 | 0.999994 | 0.999994 |  |
| *psbL* | 0.19247709 | 0.017559784 | 0.016945906 |  |
| *psbM* | 0.15258333 | 0.999398181 | 0.999978 |  |
| *psbN* | 0.99999994 | 0.999905005 | 0.999955001 |  |
| *psbT* | 0.59464203 | 0.831337026 | 0.828372787 |  |
| *psbZ* | 1 | 0.999976 | 0.999995 |  |
| *rbcL* | 5.25E-40 | 5.27E-21 | 5.20E-21 | 28D,33D,91P,149Q,219V,279T,326V,424V,449A |
| *rpl14* | 1 | 0.999930002 | 0.981298092 |  |
| *rpl16* | 7.13E-26 | 9.11E-17 | 1.48E-18 | 119S |
| *rpl20* | 1 | 0.999995 | 0.999995 |  |
| *rpl22* | 1 | 0.999999 | 0.999997 |  |
| *rpl23* | 0.7710184 | 1 | 0.9996935 |  |
| *rpl2* | 1.89E-17 | 2.20E-10 | 1.66E-10 | 166H,168P |
| *rpl32* | 0.11100548 | 0.450752442 | 0.417378832 |  |
| *rpl33* | 1 | 0.999375195 | 0.997018454 |  |
| *rpl36* | 0.99999988 | 0.999995 | 0.999986 |  |
| *rpoA* | 0.22574548 | 0.595542217 | 0.580071446 |  |
| *rpoB* | 1.96E-08 | 0.000250393 | 0.000117075 | 50I,801K |
| *rpoC1* | 0.0449751 | 0.497640177 | 0.426856666 |  |
| *rpoC2* | 8.13E-07 | 0.004709065 | 0.004004398 | 1364N |
| *rps11* | 1 | 0.999998 | 0.999968001 |  |
| *rps12* | 2.46E-15 | 2.64E-08 | 2.19E-10 | 116K |
| *rps14* | 1 | 0.999998 | 0.765170955 |  |
| *rps15* | 0.90348353 | 0.012879994 | 0.984341883 |  |
| *rps16* | 1 | 0.999997 | 0.999160353 |  |
| *rps18* | 0.69447474 | 0.348912536 | 0.34786041 |  |
| *rps19* | 1 | 0.999987 | 0.454852085 |  |
| *rps2* | 0.00019409 | 0.00298079 | 1.62E-05 | 12I |
| *rps3* | 1 | 0.999992 | 0.999771026 |  |
| *rps4* | 1 | 0.999905005 | 0.999946001 |  |
| *rps7* | 1 | 1 | 0.984504313 |  |
| *rps8* | 0.59203297 | 0.927542188 | 0.868876744 |  |
| *ycf1* | 5.14E-58 | 1.38E-23 | 7.00E-24 | 351V,504N,630K,631T,809L,1001C,1047F,1221Q,1224M,1225H,1235S,1384I,1387A,1389L,1503D,1531N,1596K,1735K |
| *ycf2* | 2.1546E-12 | 0.011072508 | 4.9812E-164 | 308S,657K,865S,982R,1060P,1190E,1995W |
| *ycf3* | 0.99999582 | 0.999739034 | 0.999736035 |  |
| *ycf4* | 1.1848E-07 | 6.9924E-05 | 5.58029E-05 | 58I,161L |
