# Supplementary material for: Plastome structure, phylogenomics, and divergence times of tribe Cinnamomeae (Lauraceae)
Source: BMC Genomics. 2022 Sep 8;23:642. doi: 10.1186/s12864-022-08855-4 (PMC9461114; doi:10.1186/s12864-022-08855-4)
Supplement: Supplementary file 2 — Additional file 2: Fig. S1. Comparison of the SC/IR junctions among the 15 newly generated plastomes of tribe Cinnamomeae. JLA, LSC/IRa boundary; JSA, SSC/IRa boundary; JSB, SSC/IRb boundary; JLB, LSC/IRb boundary. Fig. S2. Map of the gel electrophoresis experiments. XTBG, Cinnamomum chartophyllum; D053, C. cassia; wh020, C. longepaniculatum. Fig. S3. Structural alignment of the 15 newly generated plastomes of tribe Cinnamomeae inferred from Mauve. Fig. S4. Visualized alignments of 39 plastomes of tribe Cinnamomeae using mVISTA. The vertical scale indicates percentage of identity ranging from 50 to 100%. Exons are colored in dark blue, non-coding sequences (CNS) are colored in red, tRNA and rRNA genes (UTR) are colored in green. Fig. S5. Phylogenetic tree inferred from maximum likelihood analysis using concatenated complete plastomes with one IR removed (CP-c). Bootstrap values are indicated above branches. Fig. S6. Phylogenetic tree inferred from maximum likelihood analysis using concatenated non-protein-coding genes (NPCG-c). Bootstrap values are indicated above branches. [file 12864_2022_8855_MOESM2_ESM.zip › Additional file 2 Fig. S6.pdf]

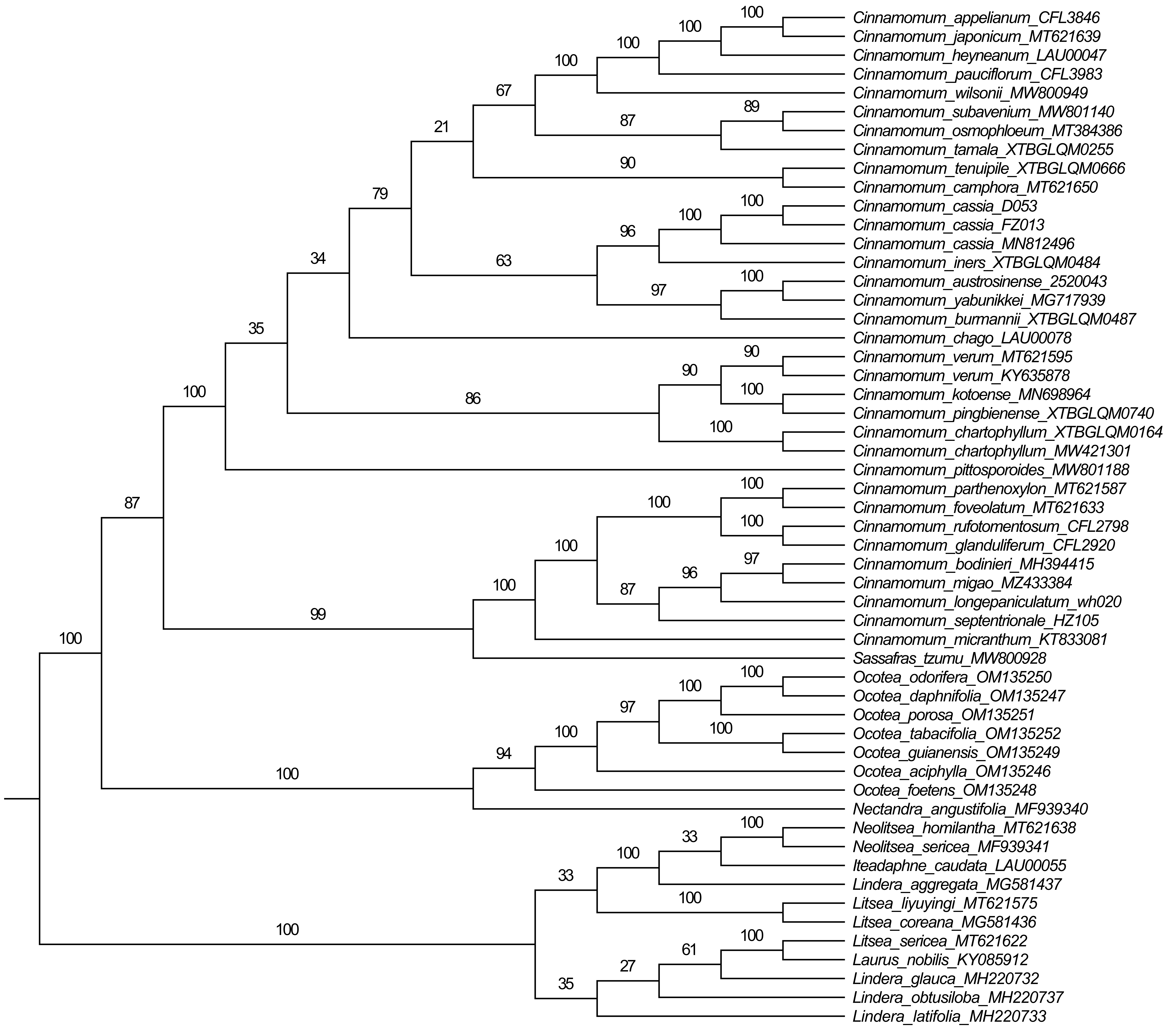

Additional file 2: Figure S6. Phylogenetic tree inferred from maximum likelihood analysis using concatenated non-protein-coding genes (NPCG-c). Bootstrap values are indicated above branches.
